# Perspective on the combined use of an independent transgenic sexing and a multifactorial reproductive sterility system to avoid resistance development against transgenic Sterile Insect Technique approaches

**DOI:** 10.1186/1471-2156-15-S2-S17

**Published:** 2014-12-01

**Authors:** Kolja N Eckermann, Stefan Dippel, Eli M Carrami, Hassan M Ahmed, Ingrid M Curril, Ernst A Wimmer

**Affiliations:** 1 Georg-August-University Göttingen, Johann-Friedrich-Blumenbach-Institute for Zoology and Anthropology, Dept. of Developmental Biology, GZMB, Ernst-Caspari-Haus, Justus-von-Liebig-Weg 11, 37077 Göttingen, Germany

**Keywords:** CRISPR, *cas9*, genetically modified insect, genetically modified organism (GMO), insect control, insecticide resistance, insect pest management, molecular entomology, quinic acid

## Abstract

**Background:**

The Sterile Insect Technique (SIT) is an accepted species-specific genetic control approach that acts as an insect birth control measure, which can be improved by biotechnological engineering to facilitate its use and widen its applicability. First transgenic insects carrying a single killing system have already been released in small scale trials. However, to evade resistance development to such transgenic approaches, completely independent ways of transgenic killing should be established and combined.

**Perspective:**

Most established transgenic sexing and reproductive sterility systems are based on the binary tTA expression system that can be suppressed by adding tetracycline to the food. However, to create 'redundant killing' an additional independent conditional expression system is required. Here we present a perspective on the use of a second food-controllable binary expression system - the inducible Q system - that could be used in combination with site-specific recombinases to generate independent transgenic killing systems. We propose the combination of an already established transgenic embryonic sexing system to meet the SIT requirement of male-only releases based on the repressible tTA system together with a redundant male-specific reproductive sterility system, which is activated by Q-system controlled site-specific recombination and is based on a spermatogenesis-specifically expressed endonuclease acting on several species-specific target sites leading to chromosome shredding.

**Conclusion:**

A combination of a completely independent transgenic sexing and a redundant reproductive male sterility system, which do not share any active components and mediate the induced lethality by completely independent processes, would meet the 'redundant killing' criteria for suppression of resistance development and could therefore be employed in large scale long-term suppression programs using biotechnologically enhanced SIT.

## Background

Many insects heavily damage agriculture and forestry or transmit deadly diseases to animals and humans. Current control efforts still mostly rely on the use of insecticides, but chemical control is not always harmless and the costs of developing new chemical compounds to overcome the world-wide threat of insecticide resistance are escalating [1]. Moreover, to protect biodiversity the establishment of pest-specific management methods is desirable. The Sterile Insect Technique (SIT) is a species-specific genetic control approach that acts as an insect birth control measure, which relies on the mass rearing, sterilization and field release of large numbers of insects. The competition between released sterile and resident males for mating with wild females leads to the reduction of the reproductive potential. If continued releases of high-quality sterile males in inundating numbers over several consecutive generations are performed, a progressive reduction of the population size and eventually the total eradication of the pest population will occur [2,3]. SIT is now an accepted component of various integrated approaches to control, suppress, prevent, or even eradicate invasive insect pest species from islands, large fruit production areas, or even complete continents [4]. Classically, both male and female insects were released, particularly because the distinction between male and female pupae is hardly manageable or requires the development of genetic sexing strains [5]. Released females, however, although sterile, sting fruits with their ovipositors or keep blood feeding and potentially transmit diseases as well as compete against wild females for mating with the sterile males [5]. In addition, sterilization is classically achieved by irradiation, a procedure that often renders insects very weak and unfit to compete with the wild mates [6]. Such drawbacks and many years of experience have put forward several key requirements for an efficient SIT application: intensive rearing of large numbers of insects for mass release, the availability of efficient sex-separation methods, sterilization techniques able to handle large numbers of insects with minimal effects on fitness and competitiveness, effective release methods, and efficient marking systems to identify released individuals during monitoring of SIT programs.

Biotechnological engineering of insects makes novel approaches possible to efficiently mark insects as well as selectively produce vigorous and potent sterile males, which are generated by conditional male reproductive sterility in combination with conditional female lethality. This will improve efficacy and widen applicability to further insect pest species [7,8]. To minimize the concerns coupled with the release of transgenic organisms, SIT programs are actually ideal, as the sterility of the released males will serve as a biological safety mechanism for containment as it impedes the spread of transgenes and allows for a safe deployment [9,10].

In accordance to this hope for novel successful genetic pest management strategies, the first biotechnologically engineered designer insects have already been released in small scale trials: pink bollworm moths in Arizona, USA [11], as well as yellow fever mosquitoes in the Grand Cayman Islands [12], Malaysia [13], with a currently ongoing release in Brazil [14,15]. For the release in the Grand Cayman Islands, it has been shown that the sustained release of transgenic mosquitos carrying a dominant lethal gene could successfully suppress a field population [16] demonstrating the great potential of transgenic SIT approaches. Envisioning the beneficial future use of genetically modified insects, the European Food Safety Authority has recently published a scientific opinion on the guidance on the environmental risk assessment of genetically modified animals including insects [17]. Since reproductive sterility based on lethality systems serves as an intrinsic containment against vertical transmission of transgenes in biotechnologically engineered SIT, its application does not present real concerns in respect to humans and the environment [18].

Nonetheless, the use of transgenic SIT approaches is still at initial stages and an ongoing large scale use somewhat premature, as potential resistance development might pose a significant threat to the further use of this technology [19]. In the currently released transgenic mosquitoes, the dominant lethality is mediated by the overexpression of a synthetic transcription factor that is deleterious to cells at very high levels reached by auto-activation in a positive feedback loop [20]. This presents just one single killing system based on an unclear mechanism. Since most pest insects produce large numbers of offspring, they have a high propensity to evolve resistance to control measures. Actually classic SIT based on sterilization by irradiation is an exception in the resistance development context, as the radiation-induced breaks of the chromosomes are random and vary among all individuals thus providing built-in redundancy [21]. However, transgenic SIT approaches with defined killing systems are in principle susceptible to resistance development. Thereby, we assume that the released insects still contain functional transgenes and are themselves susceptible to the dominant lethality [22]. The potential break down of transgenic characters during mass rearing is an additional important but different issue for quality control before release. In respect to resistance development the heterogeneous genomes of the field populations are important [21], which might contain genotypes that lead to suppression or partial suppression of the lethality traits. For the avoidance of behavioural resistance, where wild type insects reject mass-reared insects as mating partners, regular introgression of wild type genetic material into the mass rearing strains has been successful [3]. However, there is also the possibility of biochemical resistance to biotechnologically engineered lethality. Due to the inundation of the population with susceptible alleles by the release of the sterile insects during an ongoing SIT program, only strong resistance-mediating alleles acting dominant and having only low fitness costs propose a threat to SIT programs but are so far only hypothetical [22].

Nevertheless, insects have successfully developed resistance to synthetic chemicals as well as to microbial agents [23] and are also likely to develop resistance to transgenic SIT approaches when employed in long-term suppression programs [24]. One strategy to significantly impede or at least delay resistance development could be based on the principle of 'redundant killing' [25,26]. Therefore, transgenic SIT strains with effective and necessary sterility or lethality traits should only be considered in large scale long-term suppression programs, once completely independent toxicity systems have been combined. Since actually two traits are favourably introduced by transgenesis - female lethality for male only releases as well as reproductive sterility by dominant lethal transgenes - one task is to identify two completely independent ways of mediating them.

### Combination of two independent systems: male reproductive sterility and female lethality

A sterile insect in the sense of SIT is defined as "an insect that, as a result of a specific treatment, is unable to reproduce" [27]. A first approach to cause such reproductive sterility by biotechnological engineering was successfully demonstrated in the non-pest insect *D. melanogaster *[28]. The system is based on the transmission of a transgene combination that causes conditional embryo-specific lethality in the progeny without larval hatching and has successfully been transferred to tephritid fruit flies [29,30]. This prevents larval damage to fruits and the introgression of transgenes into wild type fruit fly populations. Furthermore, for tephritid fruit flies and mosquitoes, transgenic strains were produced using an autocidal overexpression loop of the protein tTA, which leads to dominant lethality when transgenic males were mated to wild type females [20,31]. Additional transgenic reproductive sterility systems [32,33] might be based on species-specific homing endonucleases [34].

**Figure 1:**
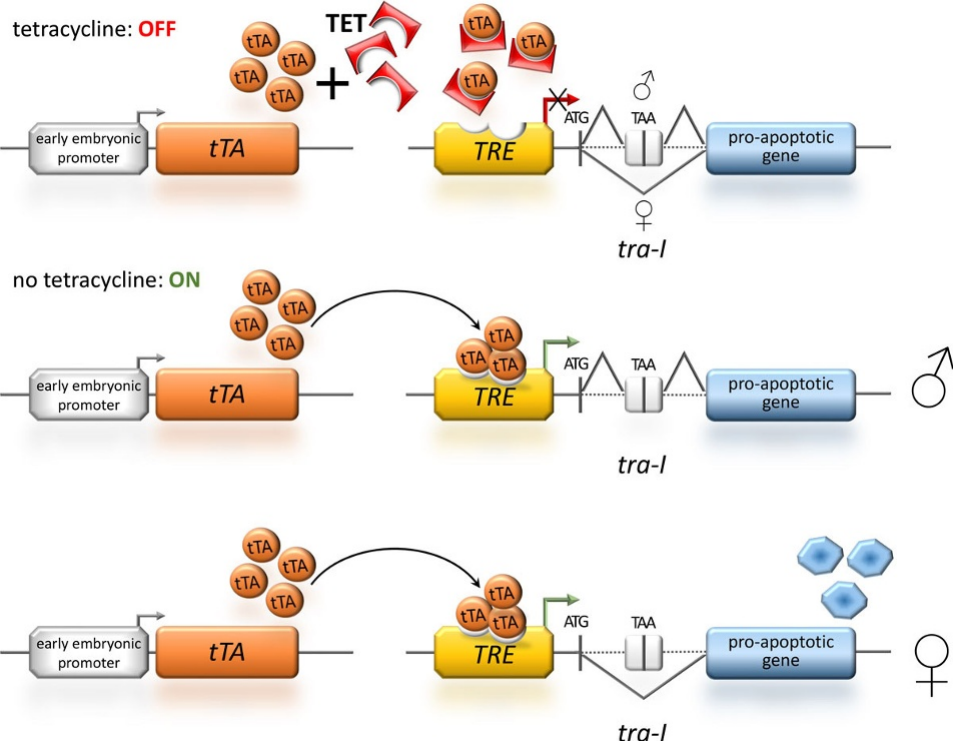
**Sexing using female-specific splicing under the control of the repressible tTA-system**. The depicted transgenic sexing system [41,42] uses a sex-specifically spliced intron and a hyperactive pro-apoptotic gene to generate female-specific lethality under the regulation of the tetracycline-controlled transactivator (tTA). To cause early embryonic lethality and thus avoidance of larval survival, the tTA is under the control of an early embryonic promoter. During rearing of such strains, addition of tetracycline (TET) to the food keeps the system in the OFF state, as tetracycline blocks the binding of tTA to its response element (*TRE*). For the release generation, tetracycline is absent in the food and therefore the sexing system is ON: in males, the male specific splicing of the transformer intron (*tra-I*) - placed after the translation start codon (ATG) of the effector gene - includes a small exon containing a TAA stop codon between the start codon and the rest of the effector gene and therefore prevents the production of the functional pro-apoptotic effector protein allowing the males to survive; whereas in the females the female specific splicing of the *tra-I *produces a functional effector and the embryonic cells are driven into apoptosis, which leads to female-specific embryonic lethality.

To generate transgenic sexing systems, female lethality was first developed and tested in *D. melanogaster *and based on the female-specific expression of conditional lethal genes [35,36]. More recently transgenic sexing systems for tephritid fruit flies have been generated using a female-specifically spliced intron from the *transformer *gene. First it was used in an autocidal expression loop with the female lethality occurring at late larval stages in the Medfly *Ceratitis capitata *[37]. This system has successfully been transferred to other Tephritids such as the olive fly *Bactrocera oleae *[38] and also to blowflies [39]*- *devastating pests of livestock - as well as to lepidopterans [40]. Furthermore, embryonic transgenic sexing systems have combined the use of such a female-specifically spliced intron with an early embryonic expression mediated by *cis*-regulatory elements from early acting cellularization genes that indirectly and controllably drive the expression of a hyper-active pro-apoptotic gene (Figure 1) [41,42]. An even better understanding of the sex differentiation pathways in insects will provide us with additional strategies for synthetic genetic-based tools for large scale sex separation in SIT applications [43] based on either female killing or actual female sex-reversal [44,45].

### tTA: the commonly used conditionally repressible expression system

The conditionality of the so far established transgenic sexing and reproductive sterility systems is based on a binary expression system, which can be suppressed by supplementing the food with tetracycline (Figure 1). The tetracycline-controlled transactivator (tTA) consists of a bacterial-viral fusion protein [46] that activates gene expression after binding to a tTA-response element (*TRE*). The major advantage of this binary expression system is that a food supplement can suppress the activation providing an additional control to the directed gene expression [47]. tTA complexed with tetracycline is prevented from binding to its response element and the downstream gene is not activated. The expression system is thus switched off by supplementing the food with tetracycline, which allows for an additional control on top of the tissue-specific promoter driving tTA expression. Since only small amounts of tetracycline are needed to suppress the expression, this system has become the most favourable expression system to develop transgenic SIT approaches. However, to create a situation of 'redundant killing' based on two completely independent mechanisms to mediate reproductive sterility and female lethality, an additional conditional expression system is necessary.

### Second food-controllable expression system: Q system

**Figure 2:**
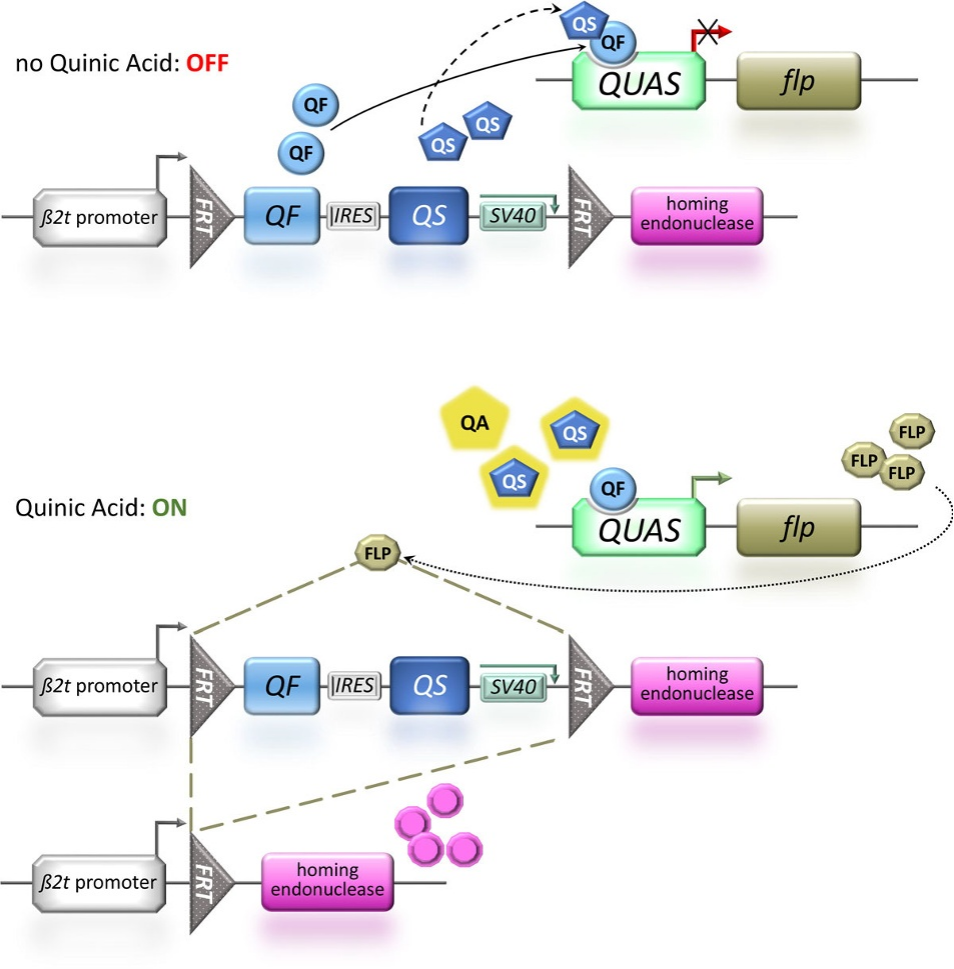
**Reproductive sterility using a homing endonuclease controlled by the inducible Q-system in combination with site-specific recombination**. The proposed reproductive sterility system is based on the inducible binary expression system Q [48], in which quinic acid (QA) acts as an inducer that hinders the repressor QS from complexing the transcriptional activator QF, which can activate its target genes by binding to a Q upstream activation sequence (*QUAS*). To generate male reproductive sterility systems the spermatogenesis-specific promoter of the *β2 tubulin (β2t) *gene can be suitably used to affect either the sperm itself or the progeny sired by the sperm. The Q system can be combined with a recombinase mediated transcription regulation system to render the induction of an effector gene expression permanent and independent of the presence of the inducer QA. In this dual system, QF drives the expression of a site-specific recombinase (FLP) that can in turn remove a *flp*-out cassette [57], which contains a transcriptional terminator (*SV40*) and is flanked by *flp *recombinant target sites (*FRT*s) in direct orientation. After the removal of the transcriptional terminator, the directed expression of an effector gene is mediated by the tissue-specific promoter 5' to the FRT. Since the Q system components are superfluous after the activation of the effector gene, they can also be placed into the *flp*-out cassette. To make sure that both components of the Q system are translated in a bi-cistronic messenger RNA, they will be separated by an internal ribosome entry site (IRES). A homing endonuclease targeting the progeny genome can be employed as an effector that would kill the progeny but not the sperm itself [34]. During regular rearing this male reproductive sterility would be kept in an OFF state, as at the absence of QA the repressor QS will mask QF and block its activation potential. Only after the addition of QA to the food in the release generation, QS will be inactivated and QF thereby allowed to activate the expression of the *flp *recombinase (FLP), which in turn would remove the Q system regulators and at the same time mediate the expression of the homing endonuclease that could block development of the next generation and thus cause male reproductive sterility.

Recently a second food-additive controllable expression system - the Q system - has been shown to work *ex vivo *in mammalian cells as well as *in vivo *in the vinegar fly *D. melanogaster *[48,49]. The broad applicability of this system is also demonstrated by its functionality in the nematode worm *Caenorhabditis elegans *[50]. The Q system is based on the regulatory genes of the gene cluster *qa *from the bread mold *Neurospora crassa*, which allows the fungus to utilize quinic acid as a carbon source [51]. Quinic acid can be found in high concentrations both in herbaceous plants as well as conifers [52] and at especially high levels in unripe fruits [53]. Several molds are able to use quinic acid as carbon source and have specific gene clusters for the catabolic pathway [54]. The regulatory genes of the cluster ensure that the catabolic enzymes are only expressed at the presence of quinic acid: one gene, *qa-1F *(*QF*), acts as DNA-binding transcriptional activator of all cluster genes, whereas another regulatory gene, *qa-1S *(QS), acts as a repressor that does not bind DNA itself but inactivates the activator QF by complex formation [54]. Quinic acid acts as an inducer by hindering the repressor QS from complexing QF, which then can activate its target genes (Figure 2). Therefore, the Q system is actually an inducible binary expression system with the food additive, quinic acid, leading to the activation of controlled gene expression. This and the fact that quinic acid is found widespread in nature [52,53] do not allow us to use this system in an analogous way to the tTA system. However, it offers a completely independent food additive-controlled expression system that should be utilized for novel transgenic SIT approaches.

### Render inducible system suitable for transgenic SIT approaches

An inducible system would usually require that the inducer is constantly present to have the system activated. But as this cannot be warranted for a food-additive after release, a temporary induction of the system needs to be stabilized into a continuous expression. For this purpose site-specific recombination systems [55] can be utilized to stabilize an inducer pulse into a persistent activation. For the *flp *recombinase (FLP), it was demonstrated in *D. melanogaster *that a region-specific promoter can be separated from the downstream coding region by a *flp*-out cassette that contains a transcriptional terminator and is flanked by *flp *recombinant target sites (FRTs) [56,57]. The transcriptional terminator prohibits the directed expression mediated by the tissue-specific promoter until FLP removes the *flp*-out cassette by site-specific recombination of the FRTs that are in direct orientation (Figure 2). The left over single FRT in the 5'UTR does not interfere with effective transcription and translation of the downstream coding sequences [56,57]. On this basis, the Q binary system can be combined with the FLP mediated transcriptional activation system to stably activate the expression of a gene after a pulse induction with an inducer (Figure 2).

To reduce the number of constructs necessary for such a complex inducible Q and immediate targeted gene expression system, actually the regulatory components of the Q system can be placed into the *flp*-out cassette (Figure 2) which will also place the Q system components under the same control as the later expressed effector gene [57]. To actually place both regulator genes - QF and QS - into the same construct, the two coding regions can be separated by an internal ribosome entry site (IRES) to allow for a bi-cistronic transcript. Depending on the translational start efficiency of the insect virus IRES compared to the actual capped mRNA [58], the QS and QF coding sequences should be placed accordingly to make sure that repressor QS will be in surplus to the activator QF.

In *D. melanogaster *it has been shown that FLP expression driven by the *β2 tubulin (β2 tub) *promoter is highly efficient to cause cassette flip-out during spermatogenesis leading to the transmission of the activated effector construct into the next generation [56,57]. Since the *β2 tub *promoter would also enable the generation of reproductive sterility systems [7], this promoter would be very suitable for such a complex system. Respective promoters have already been cloned from a number of different tephritid and mosquito species and functionally used for sperm marking purposes [59-61].

To cause reproductive sterility, finally an effector needs to be activated that either causes male sterility by sperm depletion, e.g. by expression of a cell death gene or a cell-specific toxin that is active in the cytoplasm only and has no trans-membrane movement abilities to protect adjacent tissue or predatory organisms [7,61]. However, as such sterile males would not transfer sperm to females, such females would continue to search further for sperm-providing wild type males. Therefore an effector that would kill the progeny but not the sperm would thus be much more suitable. This will allow for sperm development and transfer and therefore renders the females at least temporarily refractory to subsequent matings with wild type males. Such an effector could be a homing endonuclease (Figure 2) that does not affect spermatogenesis - thus producing functional sperm - but attacks the genome of the zygote or prevents the fusion of the male and female pro-nuclei [34]. This would serve as the best reproductive sterility mechanism as it would cause a dominant early embryonic lethality without affecting the sperm itself by stopping the development of the progeny at the very beginning. Moreover, a homing endonuclease would also be independent in its function from the proposed hyperactive pro-apoptotic gene suggested for the sexing system (Figure 1). However, it should be noted that for an applicable transgenic reproductive sterility system, 100% male sterility needs to be reached, which requires efficient *flp *recombinase repression in the absence of quinic acid and its effective induction in the presence of quinic acid as well as strong expression of a highly active homing endonuclease.

### Partial redundancy of the female lethality and reproductive sterility systems

The described female lethality and reproductive sterility systems will in fact not be fully redundant, as only the female progeny of the released males will indeed have both lethality systems working. In the male progeny only the reproductive sterility providing the homing endonuclease will be active. Thus, rare strong resistance-mediating alleles might be selected in such male progeny and potentially lead to the accumulation of both the resistance allele and the transgenic lethality allele [22]. However, in case of direct linkage between the two lethality systems, which can be achieved by transgene modification based on site-specific recombination [62], the female lethality in the following generation would severely reduce the chance of accumulation of the lethality allele and thus reduce also the selection of the resistance allele. Since only resistant males would survive, they would be outcompeted by released susceptible SIT males [22].

### Multifactorial reproductive sterility by an endonuclease causing chromosome shredding

Ideally the reproductive sterility system itself should be highly redundant to cause many different lethal mutations similar to the built-in redundancy of radiation-induced sterility [21]. To achieve this, it would be great to have a number of diverse endonucleases or endonuclease target sites causing chromosome shredding [63]. For this, we propose the employment of an endonuclease from the adaptive bacterial immune system using as essential component clustered regularly interspaced short palindromic repeats (CRISPR) [64,65], which allows bacteria to defend themselves against viruses they encountered before by recognizing and cutting the viral DNA sequences. For the human pathogen *Streptococcus pyogenes*, it could be shown that a single endonuclease, CRISPR-associated nuclease 9 (Cas9), is sufficient to cleave the target DNA [66]. Since it was shown that Cas9 can be directed to any 'protospacer' sequence followed by a protospacer-adjacent motif (PAM) that has only two required bases (NGG) [67] by using short guide RNAs (gRNAs) [68], this CRIPSR/Cas9 system has been successfully employed in many model and non-model organisms to generate gene knock-outs and genome editing [69]. Recently a feature article on this emerging technology has discussed possible uses of the CRIPSR/Cas9 system in gene drives to alter wild populations [70].

**Figure 3:**
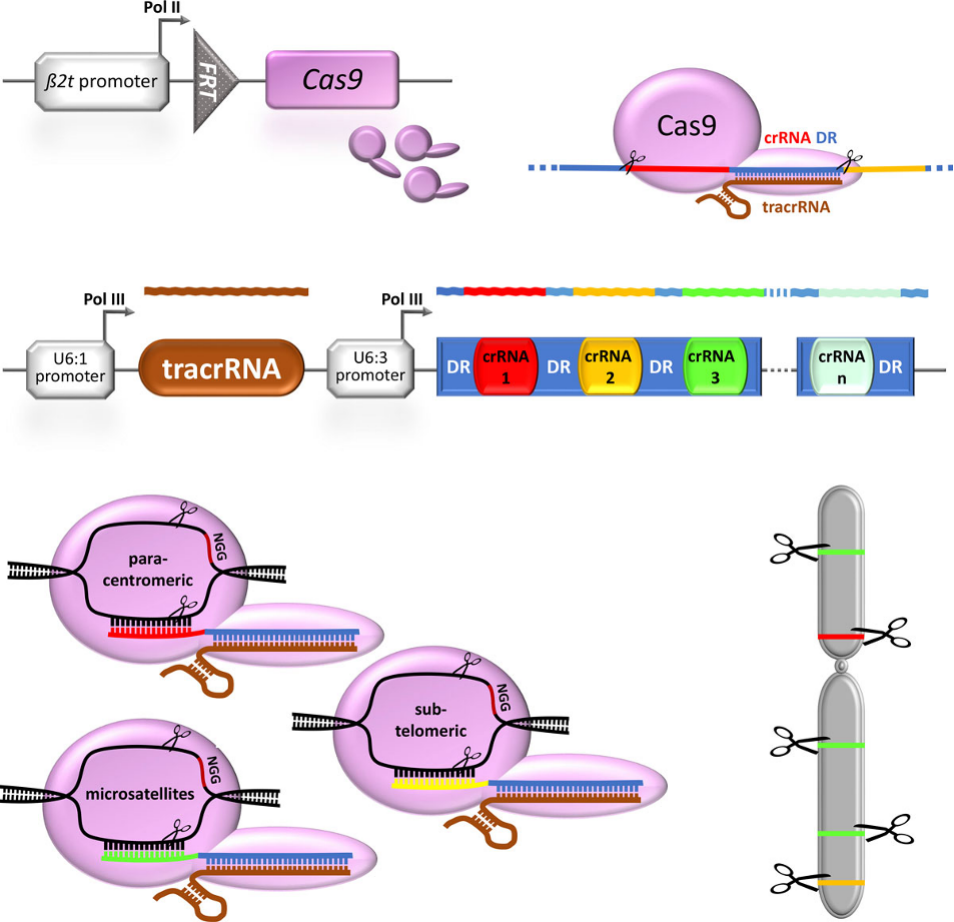
**Multifactorial reproductive sterility based on the CRISPR/Cas9 system causing chromosome shredding**. The bacterial derived Cas9 endonuclease will be expressed under the control of the *β2 tubulin (β2t) *promoter. Cas9 will be targeted to para-centromeric, sub-telomeric, and diverse macrosatellite sequences by guide RNAs, which are encoded by a CRISPR RNA (crRNA) array. This crRNA array as well as the *trans*-acting crRNA (tracrRNA) will be expressed under diverse RNA polymerase III promoters such as from the snRNA *U6 *(U6:1, U6:3). In the crRNA array, the diverse crRNAs are separated by direct repeat sequences (DR) derived from the *Streptococcus pyogenes *CRISPR. The expressed Cas9 is loaded with tracrRNA and subsequently binds the crRNA array based on complementarity between tracrRNA and the DR sequences, thereby randomly selecting one of the crRNAs as a guide to produce a functional CRISPR/Cas9 endonuclease targeting the respective genomic loci [75], which will lead to double strand breaks causing chromosome shredding.

By transgenic expression of several gRNAs using RNA polymerase III-dependent promoters, such as the *U6 *snRNA promoter, it has been shown that the Cas9 endonuclease can actually be targeted to several diverse targets, which can lead to a mutagenesis rate of up to 100% [71,72]. By our proposed use of the *β2 tub *promoter, Cas9 will be highly expressed during spermatogenesis and the mRNA still be highly translated during spermiogenesis [73] to expose the sperm chromosomes to high amounts of the endonuclease (Figure 3). To cause chromosome shredding, several guide RNAs can be employed to direct the CRISPR/Cas9 endonuclaese to para-centromeric, sub-telomeric, and microsatellite sequences. The induced double strand breaks will lead to large chromosomal aberrations causing aneuploidies that will mediate multifactorial reproductive sterility.

In fact, one of the caveats of the Cas9 technology - the potential lack of specificity leading to off-target effects [74] - can serve as an additional advantage in the proposed use here, since it might lead to pleiotropic effects harming further genomic loci. Targeting many chromosomal locations will thus provide the intended redundancy bringing the transgene-induced reproductive sterility a step closer to the built-in redundancy of radiation-induced sterility [21].

## Conclusions

The combination of a transgenic sexing system to meet the SIT requirement of male-only releases based on the repressible tTA directed expression system to create female-specific embryonic lethality using a sex-specifically spliced intron and a hyperactive pro-apoptotic gene (Figure 1) together with a reproductive sterility system based on a sperm-specifically expressed endonuclease controlled by the inducible Q-system in combination with site-specific recombination (Figure 2) seems a promising approach. These two systems would not share any active components and the lethality would be mediated by completely independent processes. Therefore, cross-resistance to both lethality-mediating processes is extremely unlikely and resistance development would require at least two independent gene loci with the likelihood of co-existence and selection being significantly reduced [25]. It should be noted, however, that this redundancy is only partial as only the female progeny of respective released males will have both lethality systems at work. While this will still reduce the likelihood of accumulating transgenic lethal alleles and resistance alleles, we propose an additional level of redundancy for the reproductive sterility system using the CRISPR/Cas9 endonuclease system targeting several chromosomal locations to induce chromosome shredding in the sperm (Figure 3).

**Figure 4:**
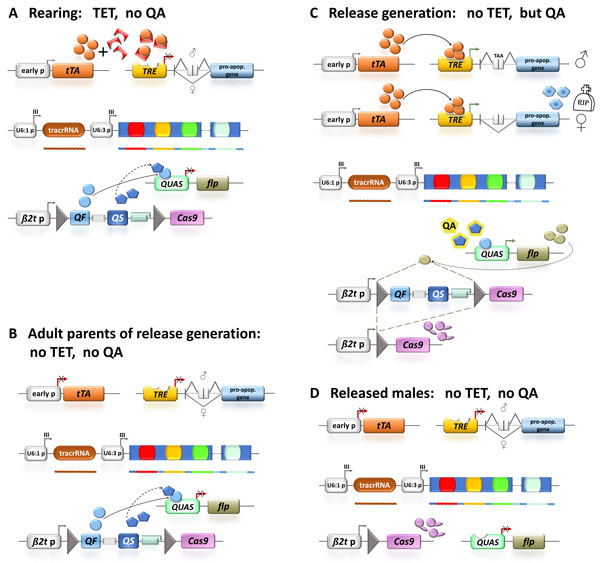
**Rearing scheme for combined female lethality and multifactorial reproductive sterility systems**. **A **Under regular rearing conditions, tetracycline (TET) is added to the food to repress the female lethality, quinic acid (QA) is not required for rearing. **B **The adult parents of the release generation will be changed to food without TET, still also without QA. This is necessary to avoid suppression of the early embryonic lethality in the next generation by maternally transferred TET to the oocyte. The female lethality system is still off, since the early embryonic promoter is not driving tTA at adult stages. **C **The release generation is then reared on food without TET but with added QA. Due to the lack of TET the female lethality system is switched on and the females die during early development. The QA leads to the activation of the Q system that leads to the expression of a site specific recombinase, which in turn mediates the spermatogenesis-specific expression of the Cas9 endonuclease by removing a recombination site-flanked spacer cassette. **D **The released males (no TET, no further QA) express high levels of the endonuclease Cas9 and multiple guide RNAs during spermatogenesis causing shredded chromosomes that will lead to lethal aneuploidy in the next generation.

The insect strains carrying the combined transgenic female lethality and multifactorial reproductive male sterility systems would be reared on tetracycline containing food to suppress the female-specific lethality. The male reproductive sterility would not be activated yet, since the repressor QS would keep the system in an OFF state (Figure 4A). The adult flies of the pre-release generation would then be aged on tetracycline-free food (Figure 4B) in order to stop the suppression of the embryonic female-specific lethality in the next generation [29,41,42]. The release generation should then be grown also on tetracycline-free larval food in order to keep the embryonic sexing system on to produce males only: in the absence of tetracycline, the synthetic transactivator tTA would activate a hyper-active pro-apoptotic gene that would lead to programmed cell death in the female embryos, as only the female-specific splicing of the *transformer *intron in this transcript results in the production of an mRNA capable of translating the functional hyper-active pro-apoptotic protein (Figure 4C). The larval food for the release generation would, however, need to contain quinic acid to inactivate the repressor QS, which would then allow the activator QF to induce the expression of the *flp *recombinase gene, which then in turn would remove the Q system regulators and mediate the expression of the heterologous endonuclease Cas9 during spermatogenesis (Figure 4C). Released males (Figure 4D) would produce sperm with shredded chromosomes leading to lethal aneuploidy in the next generation similar to radiation-induced reproductive sterility without suffering of somatic damages that cause reduced fitness.

A transgenic SIT approach using independent lethality systems would meet the 'redundant killing' criteria for suppression of resistance development and could therefore be employed in large scale long-term suppression programs.

## Competing interests

EAW holds a patent on 'Universal Markers of Transgenesis'(United States Patent No. 6,518,481 B1)

## Authors' contributions

EAW designed the project and wrote the first draft of the manuscript. KNE created the figures. All authors contributed to the conception of the project as well as critically revised and approved of the manuscript.
