# Correction to: Perspective on the combined use of an independent transgenic sexing and a multifactorial reproductive sterility system to avoid resistance development against transgenic Sterile Insect Technique approaches

**DOI:** 10.1186/s12863-022-01051-z

**Published:** 2022-05-04

**Authors:** Kolja N. Eckermann, Stefan Dippel, Eli M. Carrami, Hassan M. Ahmed, Ingrid M. Curril, Ernst A. Wimmer

**Affiliations:** Georg-August-University Göttingen, Johann-Friedrich-Blumenbach-Institute for Zoology and Anthropology, Department of Developmental Biology, GZMB, Ernst-Caspari-Haus, Justus-von-Liebig-Weg 11, 37077 Göttingen, Germany

**Correction to: BMC Genet 15, S17 (2014)**

**https://doi.org/10.1186/1471-2156-15-S2-S17**

Since the publication of this work [1], Eli M. Carrami has changed their name from Mohammad Karaminejadranjbar. Their name has been amended in the article accordingly.

## References

[1] Eckermann KN, Dippel S, KaramiNejadRanjbar M (2014). Perspective on the combined use of an independent transgenic sexing and a multifactorial reproductive sterility system to avoid resistance development against transgenic Sterile Insect Technique approaches. BMC Genet.

